# RPL35A is a key promotor involved in the development and progression of gastric cancer

**DOI:** 10.1186/s12935-021-02199-x

**Published:** 2021-09-17

**Authors:** Fang Wu, Dachuan Sun, Yuqian Liao, Kai Shang, Canrong Lu

**Affiliations:** 1 Department of Oncology, The First Affiliated Hospital of Nanchang University, Nanchang, 330006 China; 2 Senior Department of General Surgery, The First Medical Center of Chinese, PLA General Hospital, Fuxin Road, No. 28, Haidian District, Beijing, 100853 China

**Keywords:** Gastric cancer, RPL35A, Cell proliferation, Cell apoptosis, Cell migration

## Abstract

**Background:**

RPL35A has been reported to work as a biomarker in tumor angiogenesis. However, little work has been performed on the expression level and functional importance of RPL35A in gastric cancer (GC).

**Methods:**

The protein expression level of RPL35A was detected by immunohistochemical staining and western blot analysis. The Celigo cell counting assay was used to assess cell proliferation. Both the wound healing assay and the transwell assay were conducted to evaluate cell migration. Flow cytometric analysis was utilized to detect cell apoptosis and cell cycle. A mouse xenograft model was constructed for in vivo experiments.

**Results:**

The results demonstrated that RPL35A expression was abundantly up-regulated in GC and positively related to tumor infiltrate. In addition, RPL35A knockdown could significantly suppress cell proliferation, migration, enhance apoptosis and arrest cell cycle. The in vivo study also verified the inhibitory effects of RPL35A knockdown on GC tumorigenesis.

**Conclusions:**

The above mentioned results indicated that the knockdown of RPL35A might be a considerable therapeutic strategy for the treatment of gastric cancer.

**Supplementary Information:**

The online version contains supplementary material available at 10.1186/s12935-021-02199-x.

## Background

Gastric cancer (GC) is a major disease in digestive tract that seriously threatens human health with its high morbidity and mortality [1]. Currently, gastric cancer is the fifth most common cancer, and is the third leading cause of cancer-related deaths worldwide, second only to lung cancer and liver cancer [2]. Much work so far has found that the progression and prognosis of gastric cancer patients were affected by multiple factors. Taking the depth of invasion, TNM staging and lymph node metastasis rate as examples, a growing number of studies have shown that most advanced patients with GC tended to face tumor T3 or T4 diseases, extensive invasion and lymphatic metastasis [3, 4]. Admittedly, remarkable breakthroughs have been obtained in gastric cancer treatment including surgery, chemotherapy and radiotherapy. However, the clinical outcomes remain poor with a high recurrence rate, and the 5-year survival rate is less than 25% [5, 6]. Over the years, considerable efforts from collaboration of multidisciplinary team brought dawn to gastric cancer patients, achieving the shift from surgical treatment to comprehensive treatment [7]. Hence, better understanding of the molecular mechanisms of gastric cancer tumorigenesis was essential for the development of therapeutic strategies and improvement of prognosis.

Ribosomes, as is known to all, are organelles composed of a small 40S subunit and a large 60S subunit, and one of its outstanding characteristics is to catalyze protein synthesis [8]. The human L35a ribosomal protein (RPL35A), as a component of the 60S subunit, encodes a ribosomal protein. At the same time, the prior literature reported that RPL35A is located at chromosome band 3q29–qter [9]. Additionally, it was reported that RPL35A was identified as a participant in Diamond-Blackfan anemia (DBA) [10, 11], an inherited bone marrow failure syndrome, which was mainly characterized by anemia, congenital abnormalities and cancer susceptibility [12]. Besides, RPL35A, as one of the host factors, interacted with the pestivirus N-terminal protease [13]. More importantly, RPL35A played a role as a biomarker in tumor angiogenesis [14]. However, little is known about the functional roles of RPL35A in human cancers, and even less is known about the link between RPL35A with GC.

Regarding current knowledge, the strength of the present study was the first to investigate the differential expression of RPL35A in GC tumor tissues and normal tissues, confirming the mechanistic roles of RPL35A in the development and progression of GC. The data suggested that the expression level of RPL35A was abundant in GC and positively related to tumor infiltrate. We further demonstrated the effects of RPL35A knockdown on GC in vitro and in vivo, revealing that RPL35A knockdown inhibited cell proliferation and migration, promoted cell apoptosis and suppressed tumor growth. In addition, the mechanism of RPL35A regulating GC was initially explored. From the results we have obtained, one could conclude that RPL35A played a role of promotion in the development and progression of GC, which might be considered as a potential therapeutic target in GC.

## Methods

### Patients and tissue specimens

A microarray including both 110 tumor tissues and 117 normal tissues from 150 patients were purchased from Shanghai Outdo Biotech Co., Ltd., (Shanghai, China) and further analyzed. All patients who provided tissue samples signed an informed consent form. Clinical information of patients including age, gender, survival time, stage and etc*.*, was collected and recorded. Ethical approval was obtained from the First Medical Center of the PLA General Hospital.

### Cell lines and cell culture

In this study, human GC cell lines AGS and MGC-803 were purchased from Cell Resource Center, Institute of Basic Medicine, Chinese Academy of Medical Sciences (Beijing, China). Each cell line was cultured as the following conditions. In detail, AGS cells were cultured in 1640 medium with 10% fetal bovine serum (FBS). MGC-803 cells were cultured in RPMI-1640 medium 89% with 10% FBS and 1% Penicillin–Streptomycin solution. Both cell lines were maintained in an incubator containing 5% CO_2_ at 37 °C. All culture medium was changed every 3 days. We performed cell line authentication by short tandem repeat (STR) profiling and tested for mycoplasma contamination (Additional file 1).

### Immunohistochemical staining

First, gastric cancer and normal tissue microarrays were collected and dewaxed through washing with xylene and alcohol. Then the samples were subjected to antigen retrieval with 1  ×  EDTA (Beyotime Biotechnology Co., Ltd., Shanghai, China) at 100 °C for 30 min. After that, the slides were cooled to room temperature, soaked in 1  ×  PBS buffer and blocked with 3% H_2_O_2_ for 5 min and goat serum (Hengyuan Bio Co., Ltd.) for 15 min. Next, the sections were incubated with antibodies overnight at 4 °C. The detailed information of antibodies was as follows. Primary and secondary antibodies were RPL35A antibody (1:100, NOVUS) and goat anti-rabbit IgG H&L (HRP) (1:400, Abcam, Cambridge, MA, USA), respectively. Finally, the samples slides were stained with DAB for 5 min and then dyed again with hematoxylin (Baso DiagnosticsInc., Zhuhai, China) for 10–15 s. The slides were sealed and observed under an inverted microscope (IX73, Olympus, Tokyo, Japan). Two pathologists independently checked all slides at random, and then the IHC score was used for quantitative analysis. The positive cell score was graded as 0 (0%), 1 (1–25%), 2 (26–50%), 3 (51–75%), or 4 (76–100%). The staining intensity was scored as 0 (negative), 1 (weak), 2 (positive  ++) and 3 (positive  +++). IHC results based on the positive cell score * the staining intensity were classified into negative (0), positive (1–4),  ++  positive (5–8), or  +++  positive (9–12). Finally, the high and moderate expression parameters were determined by the median of IHC scores of all tissues.

### Lentivirus RNAi construction and infection

The three RNA interference target sequences were designed to construct the target gene RNA interference lentiviral vector using RPL35A gene sequence as a template. After that, the single-stranded DNA oligo containing the interference sequence was synthesized and annealed to produce double-stranded DNA. Then the double-stranded DNA was directly connected to the constructed lentiviral vector BR-V-108 through the restriction sites at both ends. The product of connection was transferred to the prepared competent E. coli cells, and then the positive recombinants were identified by PCR. Finally, AGS and MGC-803 cells in logarithmic growth phase were infected by adding 20 μL 1  ×  10^8^ TU/mL lentivirus, culturing in the corresponding medium in a 6-well dish with 2  ×  10^5^ cells per well. After 2–3 days of infection, the infection efficiency and knockdown efficiency were evaluated by fluorescence microscopy (micropublisher 3.3RTV, Olympus, Tokyo, Japan), qRT-PCR and western blot.

### RNA extraction and qRT-PCR

The cells infected lentivirus were collected and centrifuged at 2000 rpm for 5 min, then 1 mL Trizol was added for RNA extraction according to the manufacturer’s instruction of TRIzol reagent (Sigma, St. Louis, MO, USA). cDNA was obtained by reverse transcription according to the instructions of Promega M-MLV Kit (Promega Corporation, Madison, Wisconsin, USA). Real-time quantitative PCR (qRT-PCR) was performed based on the steps of SYBR Green Mastermixs Kit (Vazyme, Nanjing, Jiangsu, China). The qRT-PCR reaction volume was 10 μL, and the relative expression level of RNA was calculated by the 2^−△△Ct^ method. GAPDH was used as an internal control.

The primers sequences used in qPCR were as follows (5′–3′): The forward primer of RPL35A was 5′-GAAGGTGTTTACGCCCGAGAT-3′, the reverse primer of RPL35A was 5′-CGAGTTACTTTTCCCCAGATGAC-3′. The forward primer of GAPDH was 5′-TGACTTCAACAGCGACACCCA-3′, the reverse primer of GAPDH was 5′-CACCCTGTTGCTGTAGCCAAA-3′.

### Western blot assay

After infecting lentivirus, the cells in in logarithmic growth status were collected to extract total proteins. Twenty microgram proteins were segregated by 12% SDS-PAGE and transferred into PVDF membrane for western blot analysis. The PVDF membrane was blocked with a blocking solution (1  ×  TBST solution containing 5% skimmed milk) at room temperature for 1 h. Then, the primary antibody was added and incubated at room temperature for 2 h or overnight at 4 °C, and then washed with 1  ×  TBST solution for 3 times, 10 min each time. Thereafter, the corresponding secondary antibody was added. Finally, the chemiluminescence was evaluated by using a chemiluminescence imager.

The primary antibodies used in western blotting were as follows: RPL35A (1:2000, Abcam, #ab241070), GAPDH (1:3000, Bioworld, #60004-1-lg), JNK1  +  JNK2 (1:500, Abcam, #ab112501), p-JNK1  +  JNK2 (1:500, Abcam, #ab131499), P38 (1:1000, CST, #8690S), p-P38 (1:500, CST, #bs-5476R) and p53 (1:5000, proteintech, #60283-2-Ig). The secondary antibodies used in western blotting were Goat Anti-Rabbit (1:3000, Beyotime, #A0208) and Goat Anti-Mouse (1:3000, Beyotime, #A0216).

### HCS cell proliferation assay

AGS and MGC-803 cells with or without RPL35A knockdown were digested and resuspended into cell suspension. 100 μL cell suspension was seeded in a 96-well plate (at the cell density of 2000 cells/well) and cultured in a 37 °C incubator containing 5% CO_2_. From the second day after culturing, the cell images were taken by Celigo image cytometer (Nexcelom Bioscience, Lawrence, MA, USA). Three repetitions were set in each group. The data were performed statistical analysis and plotted the 5-day cell proliferation curve.

### Wound healing assay

AGS and MGC-803 cells were cultured in a 96-well plate (at the cell density of 5  ×  10^4^ cells/well) after the infection of shRPL35A and shCtrl. When the cell confluence was  >  90%, low-concentration FBS (Ausbian, #A11-102) was added. After that, the cell layer was scratched, and serum-free medium was added. The cells were then incubated in 0.5% FBS medium in a 37 °C incubator containing 5% CO_2_. The scratch images were graphed under a microscope at right time (0, 8, 16 or 24 h) to evaluate the migration rate of cells. The experiment was repeated three times.

### Transwell assay

First, 100 μL serum-free medium was added into the upper chamber, incubating for 1–2 h. AGS and MGC-803 cells infected with shRPL35A and shCtrl were diluted with serum-free medium at the density of 1  ×  10^5^ cells/mL and transferred to each chamber. Then, the upper chamber was transferred to the lower chamber with 600 μL medium containing 30% FBS, incubating for 48 h. After that, the cells were stained by adding 400 µL Giemsa. Finally, the cells were dissolved in 10% acetic acid and the value of OD570 was detected to analyze the migration ability of cells. The experiment was repeated three times.

### Detection of cell apoptosis and cell cycle by fluorescence activated Cells Sorting (FACS)

AGS and MGC-803 cells infected lentivirus were plated in a 6-well plate (2 mL/well). When the cell confluence reached 85%, the cell suspension was centrifuged at 1300 rmp for 5 min followed by discarding the supernatants. Then, the cells were washed with 4 °C pre-cooled D-Hanks (pH  =  7.2–7.4) and stained with Annexin V-APC (eBioscience, San Diego, CA, USA) in the dark. The cell apoptosis level was measured using FACSCalibur (BD Biosciences, San Jose, CA, USA) and the apoptotic rate was analyzed. In terms of cell cycle, AGS and MGC-803 cells infected lentivirus were plated in a 6-cm dish (5 mL/well). The qualified cells were processed as described above. Differently, the cells were washed with 4 °C pre-cooled PBS and ethanol and stained with solution PI to evaluate the changes of cell cycle by FACSCalibur (BD Biosciences, San Jose, CA, USA). Each experiment was repeated three times.

### Human Phospho-Kinase Array-Membrane

The effects of RPL35A knockdown on the phospho-kinases were detected by a Human Phospho-Kinase Array-Membrane in MGC-803 cells. The cells were lysed with 2  ×  Cell Lysis Buffer. The Handling Array membranes were blocked by 2 mL 1  ×  Wash Buffer II and incubated with cell lysates and 1  ×  Biotin-conjugated Anti-Cytokines overnight at 4 °C. Finally, the signals of membranes were determined by chemiluminescence imaging system.

### The construction of nude mouse tumor formation model

Four-week-old female BALB-c nude mice from Beijing Weitong Lihua Experimental Animals Co., Ltd., (Shanghai, China) were used in in vivo experiment. The animal experiments were approved by the Ethics Committee of the First Medical Center of the PLA General Hospital, and were in line with the Guide for Care and Use of Laboratory animals (NIH publication number 85–23, revised at 1996). Subcutaneous injection of MGC-803 cells with or without RPL35A knockdown into mice was used to construct the xenograft models, and each group contained 10 mice. The specific operation was as follows: the cells in logarithmic growth phase were digested by trypsin and suspended into cell suspension. 200 μL cell suspension (4  ×  10^6^ cells) was injected subcutaneously into mice and L and W of tumors were measured with Vernier caliper during the feeding period (L represent longest dimension and W means dimension perpendicular to length, and tumor volume was calculated as π/6  ×  L  ×  W2). The mice were sacrificed after 22 days, and the tumors were removed to weigh, and eventually frozen in liquid nitrogen and stored at − 80 °C.

### Ki-67 staining

The sections of mice tumor tissues were fixed with 4% paraformaldehyde at room temperature and 0.3% TritonX-100 was added. Then, the slides were incubated with primary antibody Ki-67 (1:200, Abcam, #ab16667) at 4 °C overnight and incubated with secondary antibody goat anti-rabbit IgG H&L (HRP) (1:400, Abcam, #ab97080) for 2 h at room temperature in the dark. Finally, the slides were stained by Hematoxylin and Eosin (Baso, Zhuhai, Guangdong, China), and observed by microscope.

### Statistical analysis

All data were analyzed by GraphPad Prism 6 (San Diego, CA, USA) and data were presented as mean  ±  SD. Statistical differences were evaluated using the unpaired t test and the value of *P* less than 0.05 was considered to be significantly different. The Mann–Whitney U analysis and the Spearman correlation analysis were used to assess the relationship between RPL35A expression and pathological characteristics of GC’s patients. All the experiments were in triplicate.

## Results

### The high expression of RPL35A in GC tissues

**Fig. 1:**
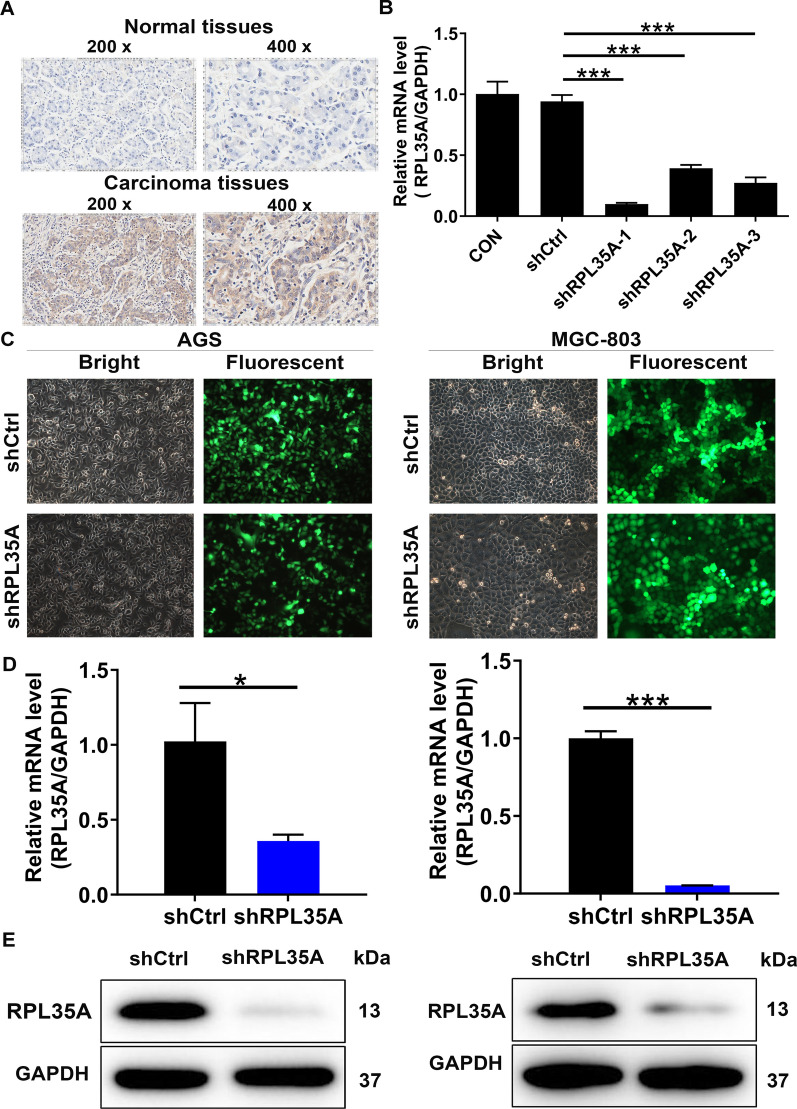
RPL35A was up-regulated in GC and RPL35A knockdown cell model was constructed. **A** The expression levels of RPL35A in GC tumor tissues and para-carcinoma tissues were determined by immunohistochemical staining. **B** The knockdown efficiencies of shRPL35A-1, shRPL35A-2 and shRPL35A-3 were detected by qRT-PCR. **C** The fluorescence expression in cells was observed after 72 h-infection. Magnification times: 200 ×. **D** The RPL35A expression in GC cell lines after infection was analyzed by qRT-PCR. **E** The expression of RPL35A protein in GC cell lines after infection was detected by western blot. Results were presented as mean  ±  SD. *P  <  0.05, ***P  <  0.001

To investigate the expression level of RPL35A in GC, a set of tissue microarrays were screened including 110 cases of GC samples and 117 cases of normal samples. Among them, RPL35A was highly expressed in 40.9% of GC tumor tissues, while in only 2.6% of normal tissues (Table 1, *P * <  0.001), which could be observed in Fig. 1A as well. Additionally, it could be revealed in the Mann–Whitney U analysis that there was a significant correlation between RPL35A expression with tumor infiltrate (*P*  <  0.05, Table 2). Furthermore, the Spearman correlation analysis further demonstrated the fact that the rises in the degree of infiltration were parallel to the increases in RPL35A expression (*P*  <  0.05, Table 3). Given the abovementioned results, we speculated that high levels of RPL35A expression might be linked to the development and progression of GC.

**Table 1:** Expression patterns of RPL35A in gastric cancer tissues and para-carcinoma tissues revealed in immunohistochemistry analysis

RPL35A expression	Tumor tissue		Para-carcinoma tissue		*P* value
	Cases	Percentage (%)	Cases	Percentage (%)	
Low	65	59.1	114	97.4	< 0.001
High	45	40.9	3	2.6

**Table 2:** Relationship between RPL35A expression and tumor characteristics in patients with gastric cancer

Features	No. of patients	RPL35A expression		*P* value
		Low	High	
All patients	110	65	45	
Age (years)				0.070
< 64	53	36	17	
≥ 64	57	29	28	
Gender				0.869
Male	84	50	34	
Female	26	15	11	
T Infiltrate				0.042
T1	5	4	1	
T2	17	11	6	
T3	65	41	24	
T4	23	9	14	
Lymphatic metastasis (N)				0.754
N0	20	13	7	
N1	18	12	6	
N2	26	12	14	
N3	46	28	18	
Stage				0.631
I	9	5	4	
II	32	21	11	
III	68	38	30	
IV	1	1	0	
Tumor size				0.591
< 5 cm	46	26	20	
≥ 5 cm	55	34	21	
Vessel carcinoma embolus				0.539
0	26	14	12	
1	54	33	21	
Nerve tumor infiltrates				0.425
0	36	22	14	
1	20	10	10	
Expession of Ki67				0.404
< 60%	47	25	22	
≥ 60%	52	32	20	
Expession of CD34				0.346
No	15	6	9	
Yes	29	16	13	
Expession of EGFR				0.531
No	85	47	38	
Yes	14	9	5	
Expession of VEGF				0.343
No	34	17	17	
Yes	65	39	26	
Expession of CDX2				0.892
No	18	10	8	
Yes	82	47	35	
Expession of Her2				0.948
No	72	41	31	
Yes	26	15	11

**Table 3:** Relationship between RPL35A expression and tumor characteristics in patients with gastric cancer

	RPL35A
Tumor infiltrate	
Spearman correlation	0.195
Signification (double-tailed)	0.041
N	110

### Construction of RPL35A knockdown cell models

Thereafter, the cell models with RPL35A knockdown were constructed to verify whether RPL35A was a participant in the development and progression of GC. First, three lentivirus-mediated short hairpin RPL35A interferences shRPL35A-1, shRPL35A-2 and shRPL35A-3 were prepared to silence RPL35A expression and screen the most effective shRPL35A by qRT-PCR, which was suggested in MGC-803 cells with five days infection. It followed that the highest knockdown efficiency was obtained in shRPL35A-1 group, reaching 89.4% (*P * <  0.001, Fig. 1B). In addition, the infection efficiencies of shRPL35A-1 in AGS and MGC-803 cells both reached  >  80% based on the green fluorescent protein (GFP) inside the cells (Fig. 1C). Subsequently, the knockdown efficiencies of shRPL35A-1 were further evaluated in the above two cell lines. The RPL35A expression was reduced by 65.0% (*P * <  0.001) and 94.7% (*P*  <  0.01) in shRPL35A group of AGS and MGC-803 cells, respectively (Fig. 1D). This was consistent with the observation from the western blot assay, indicating the down-regulated RPL35A protein levels (Fig. 1E). The above data denoted that RPL35A knockdown cell models were successfully constructed and could be utilized for subsequent experiments.

### *Silencing of RPL35A inhibited cell proliferation and migration *in vitro

**Fig. 2:**
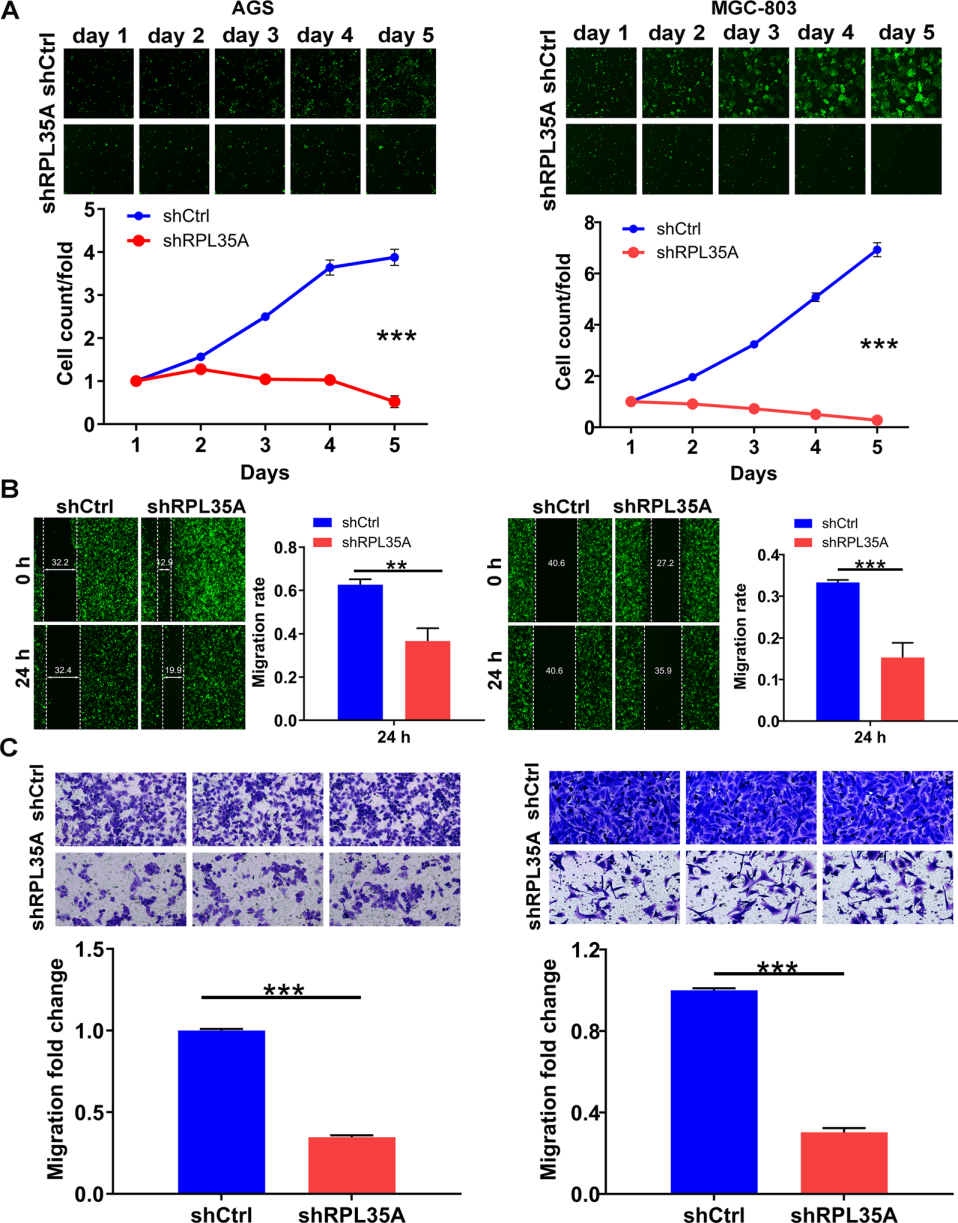
RPL35A knockdown inhibited cell proliferation and migration. **A** The cell proliferation rate was evaluated in GC cell lines after infection by the Celigo cell counting assay. **B** The migration rate of cells was detected in GC cell lines after infection by wound-healing assay. **C** The migration rate of cells was detected in GC cell lines after infection by transwell assay. Results were presented as mean  ±  SD. **P  <  0.01, ***P  <  0.001

Consequently, the effects of RPL35A knockdown on the cell phenotypes of AGS and MGC-803 cells were determined by the Celigo cell counting assay, the wound-healing assay and the transwell assay. Compared with the shCtrl group, the shRPL35A group trended to possess slower proliferation rate (*P*  <  0.001; Fig. 2A). In addition, a tendency towards lower migration rate was seen in the shRPL35A group. In detail, the cell migration rate was decreased by 42% in AGS cells (24 h), and 55% in MGC-803 cells (24 h), respectively (*P * <  0.01 for AGS cells; *P*  <  0.001 for MGC-803 cells, Fig. 2B). Identical results were obtained in the transwell assay where the migration abilities of the cells in shRPL35A group were restrained (*P * <  0.001; Fig. 2C). Taking the above observations into account, we concluded that silencing RPL35A inhibited the proliferation and migration of GC cells.

### RPL35A depletion promoted GC cell apoptosis and arrested cell cycle

**Fig. 3:**
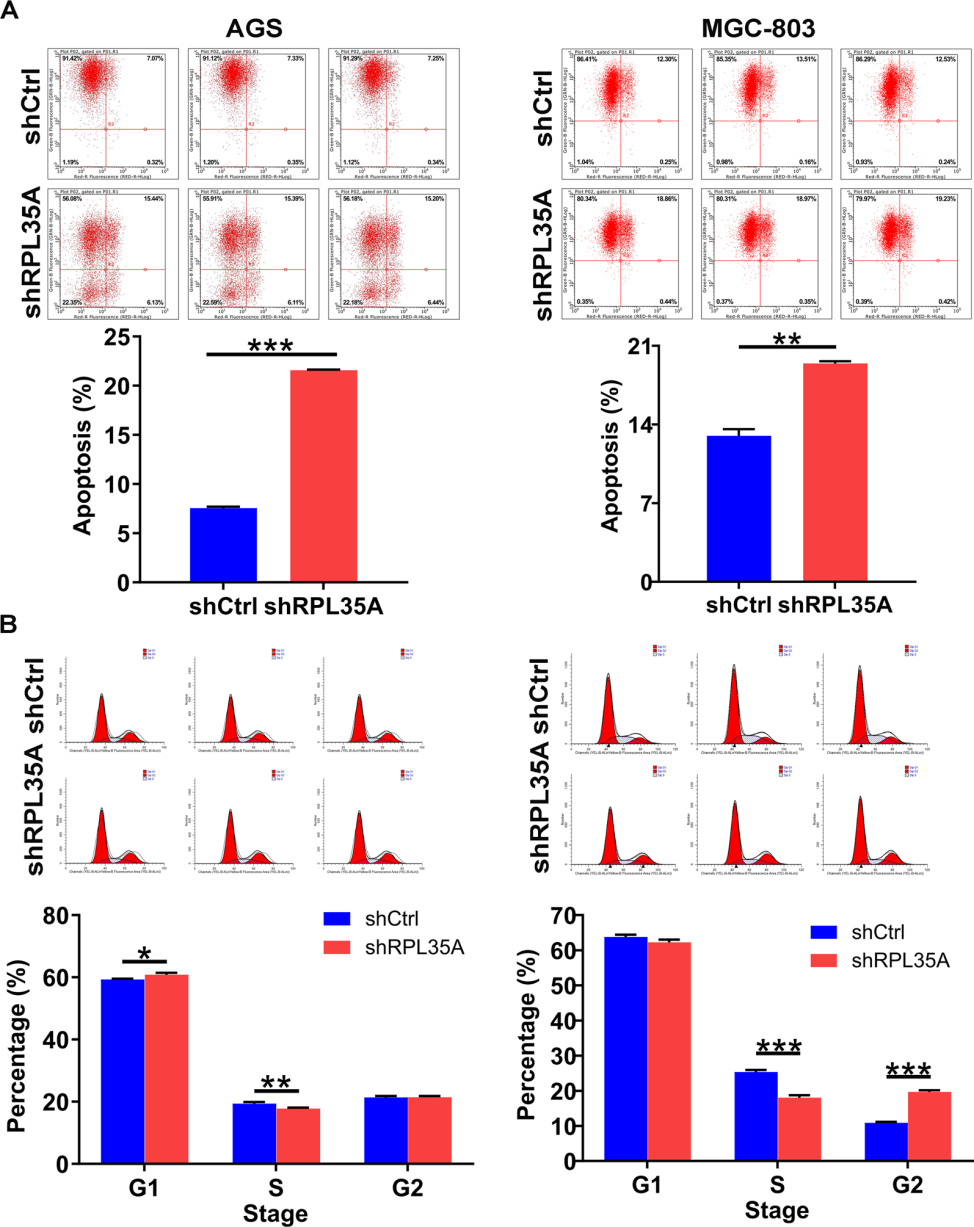
The effects of RPL35A knockdown on cell apoptosis and cell cycle. **A** The effects of RPL35A knockdown on cell apoptosis were examined by flow cytometry. **B** The effects of RPL35A knockdown on cell cycle were determined by flow cytometry. Results were presented as mean  ±  SD. **P  <  0.01, ***P  <  0.001

**Fig. 4:**
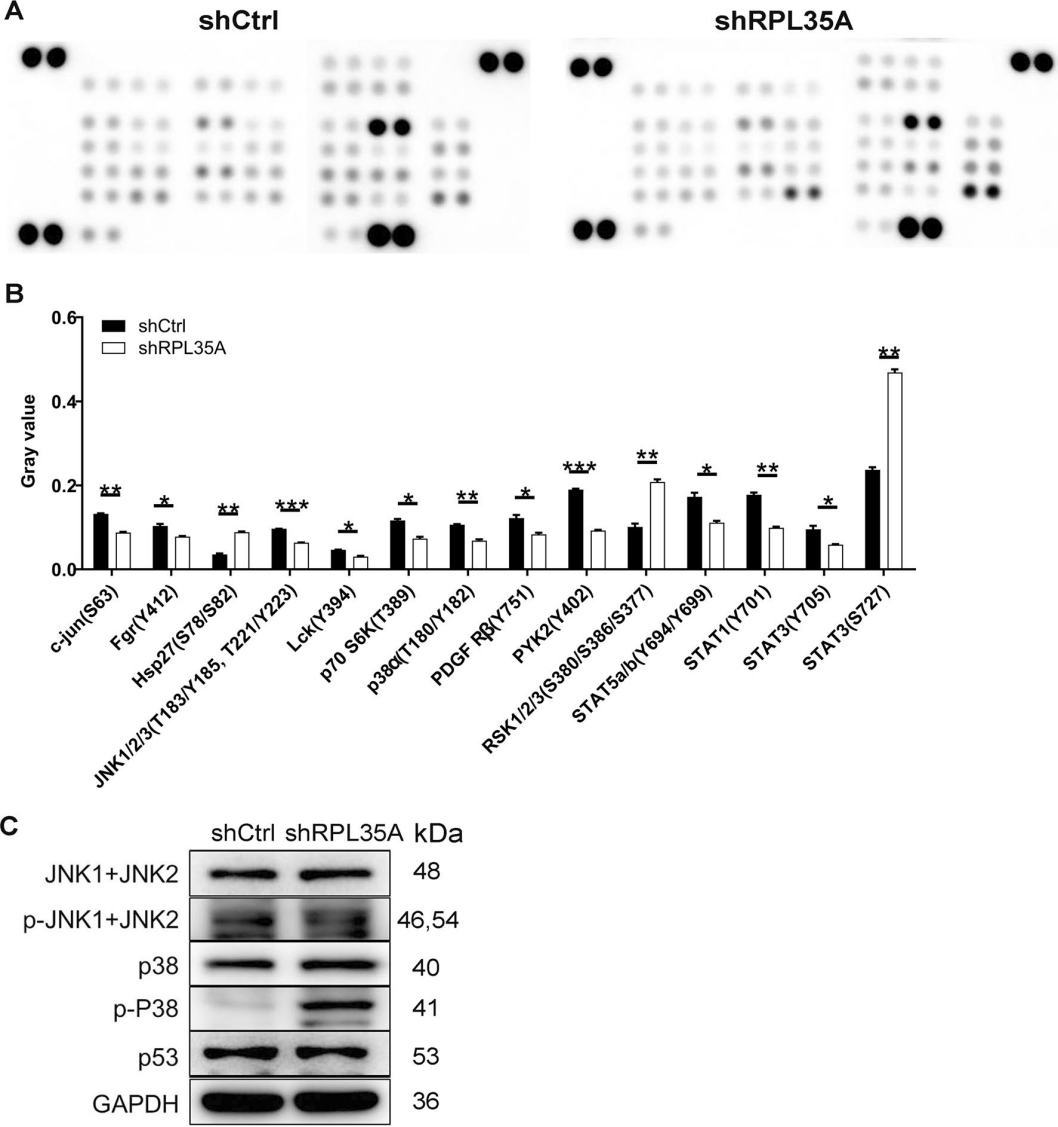
The mechanism of RPL35A regulating GC was investigated. **A** The expression of phospho-kinases in MGC-803 cells infected with shCtrl and shRPL35A was measured by ECL with Human Phospho-Kinase Array-Membrane. **B** Protein expression was presented in gray value. **C** The expression levels of several phospho-kinases-related proteins were analyzed in MGC-803 cells with shCtrl and shRPL35A by western blot. Results were presented as mean  ±  SD. *P  <  0.05, **P  <  0.01, ***P  <  0.001

What contributed to the limited cell growth might be the impaired cell cycle progression and/or increased cell apoptosis. To shed light on this hypothesis, in this regard, we analyzed cell cycle and apoptosis in RPL35A silenced GC cells by flow cytometry. It could be visualized in Fig. 3A that RPL35A depletion brought about a remarkable acceleration in cell apoptosis of AGS and MGC-803 cells (*P * <  0.001 for AGS cells, *P*  <  0.01 for MGC-803 cells). Furthermore, RPL35A suppression in the above both cells gave rise to a reduced cell population in S phase, followed by a greater arrest in G2 phase (Fig. 3B). To preliminarily investigate the mechanisms by which RPL35A regulates GC cell phenotypes, we next analyzed Phospho-Kinases given that they are critical participants in cell growth, apoptosis and metastasis. First, we analyzed alterations in the levels of 39 phospho (p)-kinases using Human Phospho-Kinase Array-Membrane. Our data showed that RPL35A knockdown changed the levels of multiple phospho-kinases, including the up-regulation of Hsp27 (S78/S82), RSK1/2/3 (S380/S386/S377) and STAT3 (S727), as well as the down-regulation of c-jun (S63), Fgr (Y412), JNK1/2/3 (T183/Y185, T221/Y223), Lck (Y394), p70 S6K (T389), p38α (T180/Y182), PDGF Rβ (Y751), PYK2 (Y402), STAT5a/b (Y694/Y699), STAT1 (Y701) and STAT3 (Y705) (*P*  <  0.05; Fig. 4A, B). At the molecular level, we consistently found that RPL35A knockdown dramatically down-regulated p-JNK1 + JNK2, while elevated p-P38 (Fig. 4C). Based on the above data, we proposed that RPL35A could modulate the levels of these phospho-kinases and in turn regulate the malignant behaviors of GC cells.

### *RPL35A depletion suppressed GC tumor growth *in vivo

**Fig. 5:**
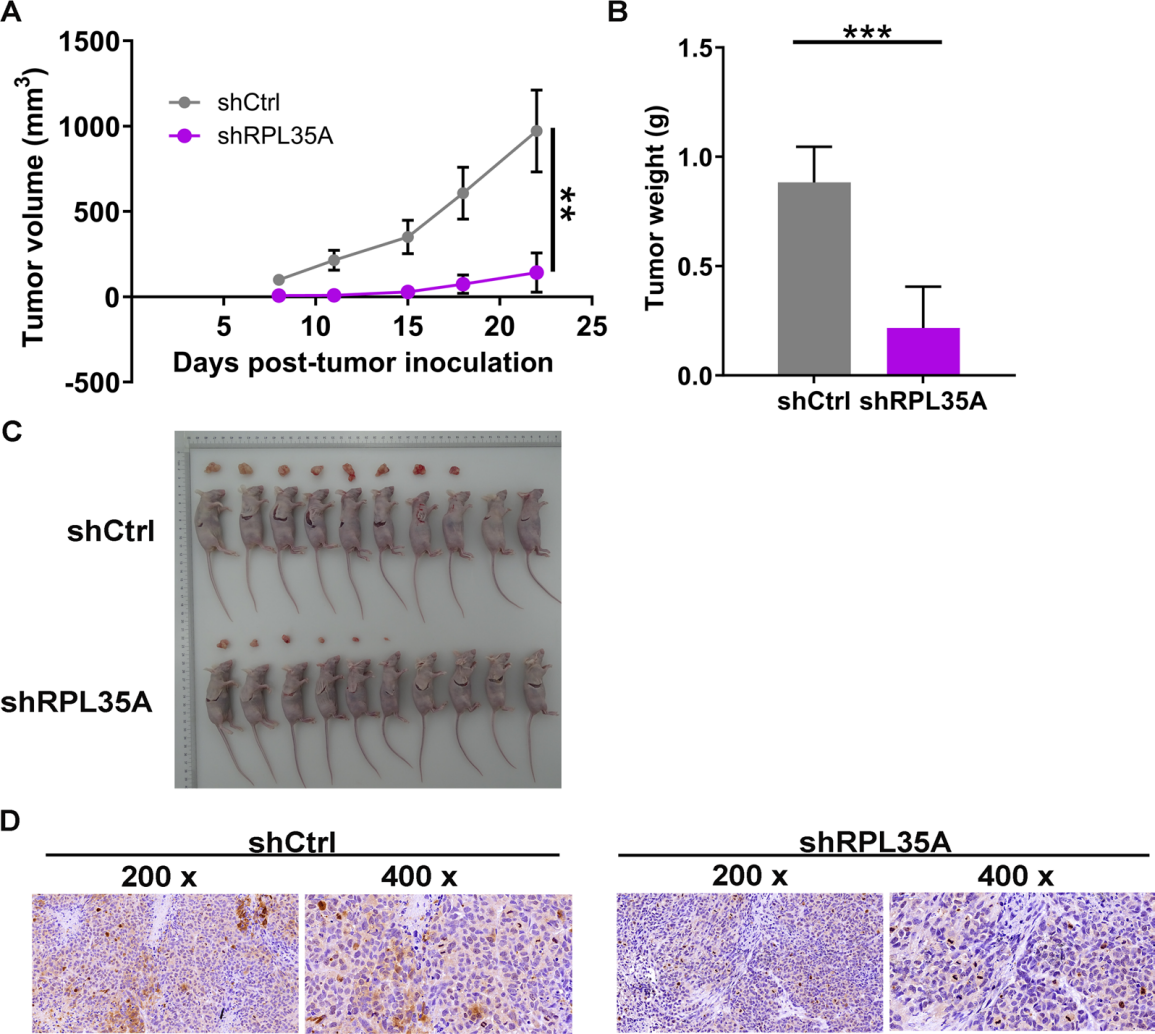
RPL35A knockdown suppressed GC growth in vivo. **A** L and W of tumors were recorded to calculate the volume of tumors from feeding to sacrifice. **B** The weight of tumors was measured after sacrificing mice. **C** The photograph of tumors was taken after removing tumors. **D** The value of Ki-67 was detected by IHC in tumor sections. Results were presented as mean  ±  SD. **P  <  0.01, ***P  <  0.001

To further confirm the effects of RPL35A knockdown on GC tumor growth, the xenograft models were constructed by subcutaneously injecting MGC-803 cells infected with shCtrl and shRPL35A. The results demonstrated that in the subcutaneous injection models, RPL35A knockdown significantly decreased the tumor volume of MGC-803 cells, which reflected that tumor growth was impaired (*P * <  0.01; Fig. 5A). Besides, mice were euthanized after 22 days using pentobarbital (100 mg/kg, P3761, Sigma, St. Louis, MO). The tumors were excised and then subjected to photograph and weigh. As a result, the weight of tumors was lighter in shRPL35A group (*P*  <  0.001; Fig. 5B, C). What’s more, the level of Ki-67, a biomarker of cell proliferation, was much lower in shRPL35A group (Fig. 5D). On the basis of our findings, it could be concluded that depleting RPL35A had an inhibitory effect on GC cell tumorigenicity in vivo.

## Discussion

Gastric cancer is a common aggressive malignancy in digestive tract characterized by an extremely high incidence and death rate [1]. Currently, despite great progress has been made in surgical resection and comprehensive treatment for GC, the prognosis of GC patients is still far from satisfactory [5]. To make matters worse, therapy resistance often exists during the treatment of advanced gastric cancer [6]. In the past several decades, targeted therapy has brought hope to patients with advanced gastric cancer to some extent and improved the overall survival rate of GC patients [15]. Therefore, exploring potential therapeutic target of GC may promote the development of molecular targeted therapy, and has become a focus in gastric cancer research. Here, we provided the first evidence that RPL35A was involved in the development and progression of GC, which might act as a promising therapeutic target for GC treatment.

RPL35A is known for its involvement in catalyzing protein synthesis [9]. Previous reports demonstrated that RPL35A was a tumor angiogenic marker [16]. Furthermore, lncNB1 binding with RPL35 contributed to the E2F1 protein synthesis, the N-Myc protein stability and the N-Myc-driven oncogenesis [17]. In the current study, we confirmed the potential tumor promotion role of RPL35A in GC and evaluated the potential link between RPL35A and GC. The results showed that RPL35A was abundantly up-regulated in GC tissues and that RPL35A expression was correlated with tumor infiltrate. Functionally, RPL35A knockdown in GC cells inhibited cell proliferation and migration, arrested cell cycle progression, and promoted cell apoptosis in vitro. Mechanically, we performed a Human Phospho-Kinase Array-Membrane in MGC-803 cells, finding the up-regulation of Hsp27 (S78/S82), RSK1/2/3 (S380/S386/S377) and STAT3 (S727), as well as the down-regulation of c-jun (S63), Fgr (Y412), JNK1/2/3 (T183/Y185, T221/Y223), Lck (Y394), p70 S6K (T389), p38α (T180/Y182), PDGF Rβ (Y751), PYK2 (Y402), STAT5a/b (Y694/Y699), STAT1 (Y701) and STAT3 (Y705) upon silencing RPL35A. More intriguingly, we found that RPL35A knockdown caused p-JNK1  +  JNK2 decreased and p-P38 increased. Importantly, RPL35A knockdown consistently inhibited GC tumorigenesis in vivo. Collectively, these results revealed that RPL35A knockdown inhibited GC growth.

Jun N-terminal kinase (JNK) signaling pathway, composed of JNK1, JNK2 and JNK3, is involved in various physiological processes such as inflammatory responses, cell differentiation, cell proliferation, cell death, cell survival and expression of proteins [18]. It was reported that deregulation of JNK is related to multiple human diseases and JNK has been identified as a novel and promising therapeutic targets for various biological diseases including cancer [19–21]. In the current study, we found that upon knocking down RPL35A, the level of p-JNK1  +  JNK2 was down-regulated. Another thing to pay attention to was the elevation of P38 phosphorylation level. P38 is identified as a multitasking kinase given that they mediate cell proliferation and migration, differentiation, stress response, cell apoptosis and survival via interacting with a plethora of substrates [22, 23]. A previous study on the evaluation of p38 MAPK inhibitors (SB203580) by Pranteda et al*.* [24] demonstrated the exact role of the p38 MAPK pathway in response to currently available colorectal cancer therapies, mainly by enhancing the phosphorylation and total proteins of p38. Furthermore, berberine might be a promising drug candidate for gastric cancer, considering that berberine, an important plant secondary metabolite, inhibited the migration and invasion of gastric cancer cells through blocking the JNK/p38 signaling pathway [25]. Collectively, we proposed that RPL35A might promote gastric cancer progression through p38/JNK signaling pathway, which indeed needs more research to support.

In conclusion, these results indicated that RPL35A played a significant role as a tumor promotor in GC and represented a promising therapeutic target for the treatment of this aggressive disease.

## Supplementary Information

**Additional file 1.** STR profiling of AGS and MGC-803 cells.

## Data Availability

Not applicable.
